# Walking ability and functional status after post-acute care for stroke rehabilitation in different age groups: a prospective study based on propensity score matching

**DOI:** 10.18632/aging.103288

**Published:** 2020-06-01

**Authors:** Chung-Yuan Wang, Seido Miyoshi, Chang-Hung Chen, Kai-Chun Lee, Long-Chung Chang, Jo-Hsuan Chung, Hon-Yi Shi

**Affiliations:** 1 Department of Physical Medicine and Rehabilitation, Pingtung Christian Hospital, Pingtung, Taiwan; 2 Department of Beauty Science, Meiho University, Pingtung, Taiwan; 3 Department of Rehabilitation, Asagi Hospital, Fukuoka, Japan; 4 Department of Neurology, Pingtung Christian Hospital, Pingtung, Taiwan; 5 Superintendent Office, Pingtung Christian Hospital, Pingtung, Taiwan; 6 Department of Healthcare Administration and Medical Informatics, Kaohsiung Medical University, Kaohsiung, Taiwan; 7 Department of Business Management, National Sun Yat-sen University, Kaohsiung, Taiwan; 8 Department of Medical Research, Kaohsiung Medical University Hospital, Kaohsiung, Taiwan; 9 Department of Medical Research, China Medical University Hospital, China Medical University, Taichung, Taiwan

**Keywords:** stroke, post-acute care, rehabilitation, cross-education, geriatric

## Abstract

Few studies have compared how rehabilitative post-acute care affects recovery of walking ability and other functions after stroke in different age groups. After propensity score matching (1:1), 316 stroke patients were separated into an aged group (age ≥65 years, n=158) and a non-aged group (age <65 years, n=158). Both groups significantly improved in Barthel index, EuroQol-5 dimension, Berg balance scale, 6-minute walking distance and 5-meter walking speed (*P*<0.001). The non-aged group had significantly larger improvements in Berg balance scale, instrumental activities of daily living, EuroQol-5 dimension and 6-minute walking distance (*P*<0.001) compared to the aged group. The two groups did not significantly differ in Barthel index, 5-meter walking speed, length of stay, and cost. The aged group had poorer walking ability and poorer instrumental activities of daily living compared to the non-aged group. After intensive rehabilitative post-acute care, however, the aged group improved in walking ability, functional performance and mental health. Intensive strength training for unaffected lower limbs in the stroke patients achieved good recovery of walking ability and other functions. Overall, intensive rehabilitative post-acute care improved self-care ability and decreased informal care costs. Rehabilitative PAC under per-diem reimbursement is efficient and economical for stroke patients in an aging society.

## INTRODUCTION

Stroke is the leading cause of adult disability [1]. A common consequence of stroke is muscle weakness, which can substantially decrease physical activity and social participation [2, 3]. Decreasing in-hospital mortality of stroke patients has increased the proportion of stroke patients who have an ambulatory disability at discharge [4]. Reduction of disability after stroke is a more informative predictor of long-time survival compared to initial disability status [5]. For stroke patients and their families, achieving independence in activities of daily living (ADL) is often the main concern [6]. For these patients, recovery of walking ability is particularly important because it is often essential for ADL and increases the likelihood of discharge [7, 8]. In geriatric stroke patients, walking ability is associated with a high probability of functional recovery [9]. Although the training effect in geriatric stroke patients is generally poor, some studies have compared the impact of rehabilitative post-acute care (PAC) for stroke on functional status in different age groups [10, 11].

Since compensatory use of the unaffected limb in ADL is very common in stroke patients, achieving the goal of recovering walking ability may require balance training and limb strengthening. Therefore, rehabilitation programs aimed at restoring standing balance after stroke tend to target the compensatory role of the strong limb rather than weight bearing asymmetry [12]. “Cross-education” refers to training one side of the body to increase strength in the same muscles on the untrained side. Some cross-education studies indicate that high-intensity training to improve strength in the limb with less neurological impairment can improve bilateral strength [13, 14].

Post-acute care refers to medical care services aimed at improving functional status and decreasing the severity of disability in patients discharged from hospitalization for acute care. In the United States, the number of the patients sent to PAC facilities has increased approximately 50% in the past 15 years [15]. In recent years, reducing the duration of PAC to minimize fee-for-service payments has become a common cost savings strategy [16]. However, discharge of patients who still need institutional PAC for rehabilitation can place additional strain on their families [16]. The social and financial impacts are particularly strong in aging societies. In 2014, the Taiwan National Health Insurance Administration (NHIA) chose stroke for its first pilot program for PAC: Post-acute Care for Cerebrovascular Diseases (PAC-CVD). This program provides per-diem reimbursement but not fee-for-service. Compared to current NHIA provisions, the program provides stroke patients with more intensive care in terms of treatment frequency and duration. The PAC-CVD program has also proven effective in reducing total hospital length of stay (LOS) and medical costs [17]. In Asagi Hospital in Japan, stroke patients who received early rehabilitative PAC that included intensive strength training for the unaffected lower limb had shorter hospital LOS and better recovery of walking ability compared to stroke patients who had received the conventional rehabilitation program. Therefore, the same training principle was adopted in our study. The purpose of this study was to explore how rehabilitative PAC under per diem reimbursement affects recovery of walking ability and other functional status in geriatric stroke patients

## RESULTS

Table 1 compares the two different age groups in terms of the studied characteristics. In both groups, most patients were male and had suffered an infarction stroke. Before PSM, the non-aged group had significantly more males (*P*<0.001) and significantly more patients with intracerebral hemorrhage (*P*<0.001) compared to the aged group. After PSM, the two groups did not significantly differ in gender, stroke type, common risk factors, or MRS score. Table 2 shows that all measures of functional status significantly improved after PAC for stroke rehabilitation, including BI, IADL, EQ-5D, BBS, and 6MWD.

**Table 1:** Study characteristics before and after propensity score matching (PSM)^*^.

**Variables**		**Before PSM**			**After PSM**		
		**Aged group (n=234)**	**Non-aged group (n=228)**	**P value**	**Aged group (n=158)**	**Non-aged group (n=158)**	**P value**
		**Mean±SD/n(%)**	**Mean±SD/ n(%)**		**Mean±SD/n(%)**	**Mean±SD/n(%)**	
**Gender**	Male	137(58.5)	170(74.6)	<0.001	110(69.6)	110(69.6)	1.000
	Female	97(41.5)	58(25.4)		48(30.4)	48(30.4)	
**Stroke type**	Infarction	207(88.5)	153(67.1)	<0.001	133(84.2)	133(84.2)	1.000
	Hemorrhage	27(11.5)	75(32.9)		25(15.8)	25(15.8)	
**DM**	Yes	85(36.3)	66(28.9)	0.112	43(27.2)	49(31.0)	0.536
	No	149(63.7)	162(71.1)		115(72.8)	109(69.0)	
**Hypertension**	Yes	149(63.7)	142(62.3)	0.831	97(61.4)	100(63.3)	0.816
	No	85(36.3)	86(37.7)		61(38.6)	58(36.7)	
**Dyslipidemia**	Yes	70(29.9)	66(28.9)	0.900	48(30.4)	51(32.3)	0.808
	No	164(70.1)	162(71.1)		110(69.6)	107(67.7)	
**CAD**	Yes	31(13.2)	10(4.4)	0.001	11(7.0)	10(6.3)	1.000
	No	203(86.8)	218(95.6)		147(93.0)	148(93.7)	
**Af**	Yes	18(7.7)	8(3.5)	0.080	5(3.2)	6(3.8)	1.000
	No	216(92.3)	220(96.5)		153(96.8)	152(96.2)	
**Previous CVA**	Yes	37(15.8)	46(20.2)	0.271	27(17.1)	32(20.3)	0.564
	No	197(84.2)	182(79.8)		131(82.9)	126(79.7)	
**MRS**	2	8(3.4)	23(10.1)	0.005	8(5.1)	18(11.4)	0.123
	3	86(36.8)	63(27.6)		55(34.8)	52(32.9)	
	4	140(59.8)	142(62.3)		95(60.1)	88(55.7)	

Abbreviations: SD, standard deviation; DM, diabetes mellitus; CAD, coronary artery disease; Af, atrial fibrillation; CVA, cerebrovascular accident; MRS, modified Rankin Scale.

^*^Non-aged group is age younger than 65 years; aged group is age at least 65 years.

**Table 2:** Total score for each functional status measure before and after PAC program in two age groups^*^.

**Measures**	**Non-aged group (n=158)**				**Aged group (n=158)**			
	**Before PAC**	**After PAC**	**t value**	**P value**	**Before PAC**	**After PAC**	**t value**	**P value**
	**Mean±SD**	**Mean±SD**			**Mean±SD**	**Mean±SD**		
**Barthel index**	48.26±22.59	73.35±22.95	19.31	<0.001	41.08±22.06	64.11±24.84	17.19	<0.001
**Euro QoL-5 dimension**	10.23±2.31	7.64±2.32	15.92	<0.001	10.73±2.24	8.48±2.60	13.55	<0.001
**Lawton-Brody IADL scale**	1.78±1.11	3.41±1.63	16.32	<0.001	1.20±1.23	2.70±1.63	15.39	<0.001
**Berg Balance Scale**	18.08±16.69	39.08±16.51	17.50	<0.001	14.90±13.89	30.22±16.88	15.78	<0.001
**5-meter walking speed**	0.17±0.16	0.50±0.40	13.39	<0.001	0.14±0.15	0.45±0.39	2.54	0.012
**6-minute walk distance test**	54.56±78.96	163.33±140.98	13.20	<0.001	26.38±45.17	84.23±83.59	11.42	<0.001

Abbreviations: PAC, post-acute care; SD, standard deviation; IADL, instrumental activities of daily living scale.

^*^Non-aged group is age younger than 65 years; aged group is age at least 65 years.

Table 3 shows that, in terms of ES, walking pace assessment had the largest training effect in the two age groups, and BI had the smallest training effect in the two age groups. Results for BI and walking pace assessment did not significantly differ between the two groups. However, the effects of training on EQ-5D, IADL, BBS and 6MWD were smaller in the aged group compared to the non-aged group. The largest difference between the two groups was IADL; the smallest difference between the two groups was 6MWD. Table 4 compares medical resource utilization between the two age groups. The groups did not significantly differ in average duration of stay in acute care ward, average duration of stay in PAC ward, and costs under per-diem reimbursement.

**Table 3:** Differences in effect size (ES) in each functional status measure before and after PAC program: comparison between different age groups^*^.

**Measures**	**Non-aged group (n=158)**			**Aged group (n=158)**			**ES1-ES2 (95% CI)**
	T1^**^	T2^**^	**ES1**	T1^**^	T2^**^	**ES2**	
	**Mean±SD**	**Mean±SD**		**Mean±SD**	**Mean±SD**		
**Barthel index**	48.26±22.59	73.35±22.95	1.11	41.08±22.06	64.11±24.84	1.04	0.07 (-0.03, 0.10)
**Euro QoL-5 dimension**	10.23±2.31	7.64±2.32	-1.12	10.73±2.24	8.48±2.60	-1.00	-0.12 (-0.17, -0.06)
**Lawton-Brody IADL scale**	1.78±1.11	3.41±1.63	1.47	1.20±1.23	2.70±1.63	1.22	0.25 (0.22, 0.27)
**Berg balance scale**	18.08±16.69	39.08±16.51	1.26	14.90±13.89	30.22±16.88	1.10	0.16 (0.10, 0.22)
**5-meter walking speed**	0.17±0.16	0.50±0.40	2.06	0.14±0.15	0.45±0.39	2.07	-0.01 (-0.03, 0.01)
**6-minute walk distance test**	54.56±78.96	163.33±140.98	1.38	26.38±45.17	84.23±83.59	1.28	0.11 (0.01, 0.21)

Abbreviation: PAC, post-acute care; SD, standard deviation; IADL, instrumental activities of daily living scale.

^*^Non-aged group is age younger than 65 years; aged group is age at least 65 years.

^**^T1 is pre-rehabilitation and T2 is post-rehabilitation; non-aged group is age younger than 65 years and aged group is age at least 65 years.

**Table 4:** Comparison of medical resource utilization between different age groups^*^.

**Variables**	**Non-aged group (n=158)**		**Aged group (n=158)**		**P value**
	**Mean±SD**	**Median[IQR]**	**Mean±SD**	**Median[IQR]**	
**LOS before PAC**	11.9±6.8	8[5-12]	11.4±6.9	7 [5-11]	0.543
**LOS in PAC**	29.0±16.7	21[19-42]	26.7±16.0	21 [18-39]	0.225
**Cost in PAC**	3,052.9±1,645.3	2,234.1 [2,047.3-4,182.3]	2,826.5±1,477.1	2,213.1 [2,015.4-3,821.0]	0.199

LOS, length of stay; PAC, post-acute care; SD, standard deviation; IQR, interquartile range.

^*^Non-aged group is age younger than 65 years; aged group is age at least 65 years.

## DISCUSSION

As human life expectancy increases, researchers have begun comparing functional decline in different old age groups [18]. Although a precise universal definition of geriatric age has not been established, a person aged 65 years or older is typically referred to as ‘elderly’ [19–21]. Polypharmacy is now common in older populations with multiple morbidities and has become a public health burden [22]. Non-pharmacological interventions for improving function in older stroke survivors have also attracted the attention of researchers. Rehabilitation is primarily non-pharmacologic in nature. In older populations, rehabilitation poses unique challenges and may have limited benefits. Although both hospital-based and home-based rehabilitation have demonstrated effectiveness for improving function in stroke survivors, the cost-effectiveness of rehabilitation for stroke survivors needs further research [23–25].

Hemorrhagic stroke patients tend to be younger than infarct stroke patients [26, 27]. Additionally, hypertension is more prevalent in hemorrhagic stroke than in infarct stroke [28]. However, a recent study reported that female/male ratios for ischemic stroke risk vary with age [29]. In a young or middle-aged patient, a stroke can have a large economic impact because it causes disability before the most productive years of life. Thus, younger patients tend to be highly motivated to undergo rehabilitative PAC. The non-aged group (age below 65 years) in our study had a higher percentage of patients with hemorrhagic stroke and male gender. To prevent non-comparability between the intervention group and the comparison group from distorting estimation of the treatment effect, this study performed PSM at the patient level to compare the baseline characteristics of the two groups, which increased the robustness of the analysis. After PSM, the two groups did not significantly differ in gender, stroke type, common risk factors or MRS score.

Compared to younger patients, geriatric patients are expected to have longer hospital stays after stroke [30]. In our study, the mean duration from day of stroke onset to day of PAC ward admission was 11.9 days in the non-aged group and 11.4 days in the aged group. The mean duration of PAC ward stay was 29.0 days in the non-aged group and 26.7 days in the aged group. The two groups did not significantly differ in duration of stay in acute care ward (*P*=0.543) or in duration of stay in PAC ward (*P*=0.225). Therefore, the total LOS for stroke approximated 6 weeks. In contrast, the average LOS for inpatient rehabilitation after stroke approximates 1 month in other countries (e.g., Thailand, Ireland and Switzerland) [31]. In Japan, however, the average LOS for inpatient rehabilitation is much longer. Patients who require long-term rehabilitation in Japan might be transferred to a hospital rehabilitation program up to 150 days in duration [32]. Theoretically, the per-diem reimbursement method should reduce daily expenditures even if hospital LOS is increased [33]. However, patients and hospitals in Taiwan may be motivated to minimize LOS for several reasons. First, the Taiwan National Health Insurance system classifies a stroke that requires a hospital LOS of 30 days as a catastrophic illness, which is a self-pay exemption. Second, this national pilot project (PAC-CVD) was initially designed to decrease reimbursement after a PAC ward stay of 3 weeks. Third, insufficient reimbursement of PAC costs and limited availability of PAC beds might motivate some hospitals to discharge patients as early as possible. Fourth and most importantly, patients often experience homesickness after 1 month of hospitalization and request to be discharged. To prevent cost considerations from inappropriately reducing hospital stay, this PAC-CVD program adjusted the PAC ward reimbursement in 2015. Reimbursements for all PAC ward stays of up to 12 weeks were similar. That is, reimbursements were no longer reduced for stays longer than 3 weeks after admission.

As mentioned in the Introduction section above, the PAC-CVD program was designed to improve stroke rehabilitation efficiency by increasing the intensity, frequency, and duration of rehabilitation. In contrast, current Taiwan NHIA provisions provide reimbursement for only one rehabilitative treatment per day. Compared to fee-for-service, per-diem reimbursement provides superior service at a comparable cost. The general recommendation is to re-evaluate stroke patients for further rehabilitative PAC every 3 weeks. Patients who are not motivated to undergo further rehabilitation and those who show no improvement should be recommended for discharge. In acute stroke stage, the costs of initial hospital examination (laboratory test, brain CT and brain MRI), medication and treatment are high. In the post-acute stroke stage, the care and rehabilitation costs are a potentially huge burden. In terms of minimizing and controlling hospital costs, however, per-diem reimbursement may be better than fee-for-service reimbursement in post-acute stroke stage. Therefore, per-diem reimbursement is an efficient and economical medical reimbursement plan for intensive rehabilitative PAC [17]. Per-diem reimbursement systems have been used for mental disorders in Europe [34], limited surgical diseases in Korea [35], nursing home care, domiciliary care, and adult day health care of eligible veterans in State homes [36]. Taiwan is the first country to deliver rehabilitative PAC for stroke patients under a per-diem reimbursement system.

Our study also showed that rehabilitative PAC significantly improved BI, BBS, EQ-5D, 6MWD and 5MWS. The BI is among the oldest competing indices and is widely used to assess ADL [37]. In both groups in our study, the training effect for BI was not as large as the training effect for other functional measurements. Additionally, BI did not significantly differ between the two age groups. On the other hand, walking pace assessment had the largest training effect but did not significantly differ between the two groups. For EQ-5D, IADL, BBS and 6MWD, the training effect was smaller in the aged group compared to the non-aged group. Therefore, BI alone is insufficient for evaluating walking ability and functional status in stroke patients after comprehensive rehabilitative PAC. Post-acute care for geriatric patients should also focus on improving pain, emotional change and balance coordination.

A major objective of in-patient rehabilitative training is to maximize the independence of patients in ADL in their communities. Most studies indicate that at least 3 to 6 weeks of training is needed to recover walking ability after stroke. The training time depends on the initial severity of impairment in walking function, the severity of paresis in lower extremities, the training site, and the age of the patient. A previous community-based post-stroke population study concluded that a valid prognosis of walking function can be made in 6 weeks in patients with initially severe leg paresis or paralysis and in as little as 3 weeks in patients who initially have no/mild/moderate leg paresis [38]. A systematic review article reported that, in patients who are nonambulatory in the first month after stroke, 60% of those managed in a rehabilitation unit regain independent walking ability whereas only 39% of those managed in an acute care unit do so [7]. Another study reported that advanced age was a predictor of reduced walking ability at 6 months after stroke [39]. As in Asagi Hospital in Japan, the training principles applied in our study included early rehabilitation, uninvolved limb strengthening (i.e., strengthening of both limbs rather than only the paretic limb), and enhanced lower limb strengthening. After PAC rehabilitation, the percentage of patients without independent walking ability decreased from 59.5% (275/462) to 23.6% (109/462). Further analysis showed that, in the non-aged group, 39.5% (90/228) of patients regained walking ability, but 20.6% (47/228) of patients did not. In the aged group, 33.3% of patients (78/234) regained walking ability, but 23.9% of patients (56/234) remained unable to walk.

Early and intensive mobilization after stroke is believed to reduce the time needed to restore unassisted walking function and to improve functional recovery in ADL [31, 40, 41]. In acute stage, early rehabilitation is defined as a specialized post-stroke rehabilitation program that begins within 3 days after admission [32]. In post-acute stage, early rehabilitation minimizes the duration of hospital stay before transfer to rehabilitative PAC ward. A multicenter study reported a time-dependent effect of early rehabilitation, particularly in rehabilitation for lower limb improvement [42]. Stroke rehabilitation should focus on increasing the compensatory role of the strong lower limb [12]. The increased neural drive originating from the “untrained” motor cortex is believed to contribute to the cross-education effect [43]. The cross-education effect has been confirmed in both the upper [14, 44, 45] and lower extremities [46, 47]. Different rehabilitation techniques generally achieve similar long-term effects on motor recovery [43]. A rehabilitation program that addresses motor function, balance, and independence in ADL may be needed to achieve high levels of community mobility [48]. In Japan, an intensive post-acute stroke rehabilitation program used in Asagi Hospital achieved a better hospital LOS compared to the national average (47.5 days versus 79.6 days, respectively) and a better improvement in Functional Independence Measure score between admission and discharge compared to the national average (24.7 versus 17.4, respectively). In our study, recovery of walking ability and IADL after rehabilitative PAC was generally better in the non-aged group compared to the aged group. However, the groups achieved similar improvements in BI. Restoration of walking ability and functional status in stroke patients not only benefits the hospital by reducing the resources required for formal and informal care, but also benefits the patient by reducing LOS and by improving overall health, confidence and self-care ability.

## LIMITATIONS AND CONCLUSIONS

A noted limitation of this study is that subjects were selected for only 40 days of PAC after stroke onset. Although no patients in this study received robot-based training or high-tech gait analysis, several rehabilitation facilities were used in accordance with the ability of the patient. This study only analyzed patients treated in a single regional teaching hospital. However, the number of patients who had received PAC at this hospital after stroke was among the highest of all regional hospitals in south Taiwan. Additionally, although intensive strength training for the unaffected lower limb of the stroke patient achieved good recovery of walking ability and other functions in both our hospital and Asagi Hospital in Japan, further randomized controlled trial studies are needed to compare different training methods. Rehabilitative PAC under per-diem reimbursement is efficient and economical. Further studies are needed to compare a PAC group and a control group in other regions of Taiwan under current NHIA regulations or in other nations under different reimbursement systems.

## MATERIALS AND METHODS

### Study design and sample

**Figure 1:**
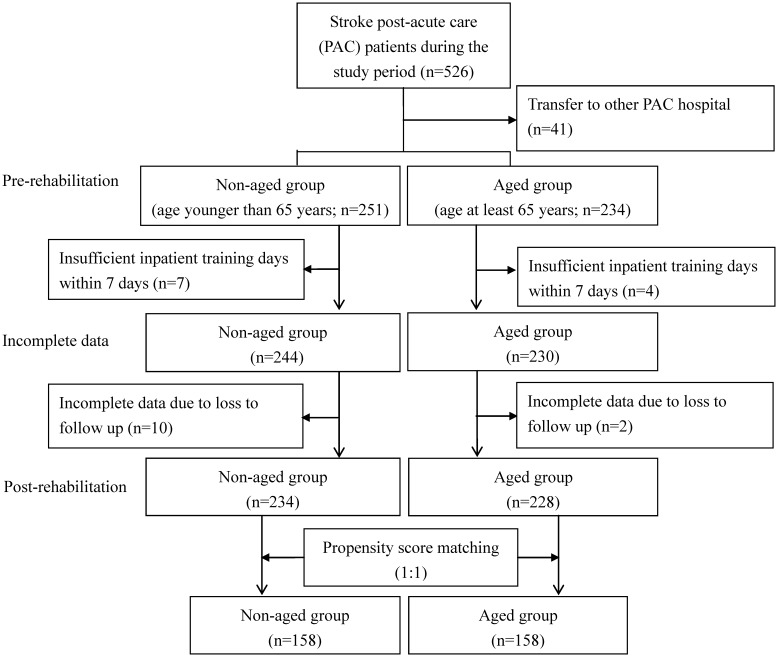
**Flowchart of recruitment and study procedure.**

The study population included all stroke patients admitted to the PAC ward at one regional teaching hospital in Taiwan between March, 2014 and May, 2019. A stroke patient was defined as a patient with a record of ICD-9-CM code 433.x, 434.x, or 436.x for ischemic stroke or a record of code 430 or 431 for hemorrhagic stroke. The inclusion criteria were (1) acute stroke; (2) admission to PAC ward within 40 days after stroke onset; and (3) Modified Rankin Scale (MRS) score of 2 to 4 where MRS scores of 0, 1, 2, 3, 4 and 5 are defined as no symptoms, no significant disability, slight disability, moderate disability, moderately severe disability, and severe disability, respectively [49]. The patients were separated into two groups: a non-aged group (patient age < 65 years) and an aged group (patient age ≥ 65 years). During the sample selection period, 526 patients were eligible for participation. Of these, 41 patients were excluded due to transfer to PAC wards at other hospitals upon request of the family or due to unavailability of PAC beds at the hospital. Another 23 patients were excluded due to insufficient inpatient training within 7 days or incomplete data. Thus, 462 patients (228 in the aged group and 234 in the non-aged group) were interviewed before and after PAC. To prevent non-comparability between the aged group and the non-aged group from distorting estimation of the treatment effect and to increase the robustness of the analysis, propensity score matching (PSM) was performed at the patient level to compare baseline characteristics of the two groups [50]. The propensity score is a balancing score that can be used to compare groups that do not systematically differ. The covariates included patient demographics (gender); clinical attributes (stroke type, hypertension, hyperlipidemia, diabetes mellitus, coronary artery disease, atrial fibrillation and previous stroke) and pre-rehabilitation functional status. The caliper matching method was used for 1:1 PSM between the aged group and the non-aged group. Thus, 158 patients in the aged group were compared with an “all participants matched set” of 158 patients in the non-aged group (Figure 1). The study protocol was approved by the institutional review board of Kaohsiung Medical University Hospital (KMUH-IRB-20140308), and informed consent was obtained from each participant before enrollment in the study.

The PAC rehabilitation programs were implemented by a multidisciplinary team comprising physiatrists, physical therapists, occupational therapists and speech therapists [17, 50]. The programs were delivered in at least three sessions per day. Each patient received 1 hour of physical therapy or occupational therapy per session. Speech therapy was arranged for patients with aphasia or dysphagia. An individualized PAC rehabilitation program was designed for each patient. The rehabilitation programs included facilitation, passive range of motion exercise, strengthening, therapeutic exercise, bed mobility training, balance training, tilt table physiotherapy, functional electrical stimulation, training under suspension, ambulation training, medical device training, transfer training, ADL functional training and coordination training. Rehabilitation of lower limb strength was enhanced by use of a cycle ergometer, treadmill, and active passive trainer and by a progressive resistance training program that included leg presses and unilateral paretic and nonparetic knee extension exercises.

### Measurement instruments

Walking ability after PAC-CVD was assessed in terms of 6-minute walking distance (6MWD) and 5-meter walking speed (5MWS). The 6MWD is a standardized assessment that is performed according to American Thoracic Society guidelines [51]. The 5MWS is recommended for assessment of longitudinal change in walking ability after stroke [52]. Barthel Index (BI), Berg Balance Scale (BBS), Lawton-Brody instrumental activities of daily living (IADL) and EuroQol- 5 Dimension (EQ-5 D) were also used to assess functional performance. The BI was used as a measure of functional disability in terms of inability to perform certain ADL [53]. The maximum score of 100 for the 10-item BI indicates complete independence. The minimum score of 0 indicates complete dependence. The 14-item BBS is a scale of functional balance [54]. Each item is rated from 0 (poor) to 4 (good), and the maximum score is 56. The IADL was used to evaluate ADL performance, including preparing food, housekeeping, laundering, making telephone calls, taking medicine, using transportation, shopping, and performing financial activities [55]. Women were scored in all eight domains while men were not scored in the domains of preparing food, housekeeping, and laundering due to cultural differences in gender roles. The EQ-5D is a self-assessment of mobility, self-care, usual activities, pain or discomfort, and anxiety or depression as part of a total health state [56]. The subject is required to score each item from 1 to 3 (no problem, some problem, and extreme problem, respectively). The Chinese versions of all instruments used in this study have been validated and used extensively in both clinical practice and research [57].

### Statistical analysis

The unit of analysis in this study was the individual stroke patient. Descriptive statistics were tabulated to depict the stroke patient demographics. Independent Sample t test and Pearson chi-square test were used to compare functional performance. Regarding total direct medical cost at hospitalization, the standard administrative claims data required by the Taiwan Bureau of National Health Insurance include fees for the following: physician, radiology, physical therapy, hospital room, pharmacy, laboratory, special materials, and others. To reflect changes in real dollar value, all dollar values were converted to their equivalent 2019 values; New Taiwan Dollar values were then converted to USD values at the average exchange rate over the 5-year period of 2014-2019.

Effect size (ES) was calculated for direct comparison of the relative magnitude of change as measured by the two functional status measures. Thus, ES was calculated as the difference between the mean scores for two time intervals divided by the standard deviation in the score for the previous time interval [58]. Using this method of standardizing the extent of change measured by an instrument enabled comparisons between two instruments. An ES of 1.0 is equivalent to a change of one standard deviation in the sample. Effect sizes of 0.2, 0.5 and 0.8 are typically considered small, medium and large changes, respectively. Differences in ES and associated 95% confidence intervals were also calculated in bias-corrected and accelerated bootstrapping with 1,000 replications [59].

Statistical analyses were performed using Stata Statistical Package, version 13.0 (Stata Corp, College Station, TX). All tests were two-sided, and *P* values less than 0.05 were considered statistically significant.
